# 90-Day all-cause mortality can be predicted following a total knee replacement: an international, network study to develop and validate a prediction model

**DOI:** 10.1007/s00167-021-06799-y

**Published:** 2021-12-06

**Authors:** Ross D. Williams, Jenna M. Reps, The OHDSI/EHDEN Knee Arthroplasty Group, Peter R. Rijnbeek, Patrick B. Ryan, Daniel Prieto-Alhambra

**Affiliations:** 1 Erasmus University Medical Centre, Rotterdam, The Netherlands; 2 Janssen Research and Development, Raritan, NJ USA; 3 NDORMS, University of Oxford, Oxford, UK

**Keywords:** Knee arthroplasty, Prediction, Mortality, Surgery, Risk model, Clinical decision aid

## Abstract

**Purpose:**

The purpose of this study was to develop and validate a prediction model for 90-day mortality following a total knee replacement (TKR). TKR is a safe and cost-effective surgical procedure for treating severe knee osteoarthritis (OA). Although complications following surgery are rare, prediction tools could help identify high-risk patients who could be targeted with preventative interventions. The aim was to develop and validate a simple model to help inform treatment choices.

**Methods:**

A mortality prediction model for knee OA patients following TKR was developed and externally validated using a US claims database and a UK general practice database. The target population consisted of patients undergoing a primary TKR for knee OA, aged ≥ 40 years and registered for ≥ 1 year before surgery. LASSO logistic regression models were developed for post-operative (90-day) mortality. A second mortality model was developed with a reduced feature set to increase interpretability and usability.

**Results:**

A total of 193,615 patients were included, with 40,950 in The Health Improvement Network (THIN) database and 152,665 in Optum. The full model predicting 90-day mortality yielded AUROC of 0.78 when trained in OPTUM and 0.70 when externally validated on THIN. The 12 variable model achieved internal AUROC of 0.77 and external AUROC of 0.71 in THIN.

**Conclusions:**

A simple prediction model based on sex, age, and 10 comorbidities that can identify patients at high risk of short-term mortality following TKR was developed that demonstrated good, robust performance. The 12-feature mortality model is easily implemented and the performance suggests it could be used to inform evidence based shared decision-making prior to surgery and targeting prophylaxis for those at high risk.

**Level of evidence:**

III.

**Supplementary Information:**

The online version contains supplementary material available at 10.1007/s00167-021-06799-y.

## Introduction

TKR surgery is generally a safe procedure with fewer than 10% of patients experiencing post-operative complications. These adverse events include short-term (e.g. 90-day) post-operative mortality [12, 15]. Mortality following TKR is low and has been declining over recent years [2]. However, there is a scarcity of data on who is at risk of post-operative death, and a related prediction tool or algorithm would help inform decisions for patients subjectively at risk of complications. For example, a high-risk patient may opt-out of surgery as the long-term benefits are outweighed by the cost. Providing a short-term mortality risk model could help inform decision making regarding whether to opt for the surgery and to help target preventative interventions.

To be clinically useful, covariates included in any model must be readily available at the time of model implementation. For this study this means pre-operatively. Current prediction model studies of post-operative outcomes after TKR have several limitations. In a recent review predicting post-operative infection after total joint replacement [9], most models were not externally validated, the process of applying a model in a new database to check if performance transfers to new data, and none were ready for clinical use due to issues with application (e.g. variables unobtainable at time of use) or insufficient performance. Some models were developed using data that were not routinely collected in observational data (e.g., floor of a patient’s bedroom, preoperative walking distance) and therefore validation of these models was infeasible using the data available in this study. Finally, most models had not taken full advantage of all data available in medical records. For example, using a comorbidity index [6] instead of all patient characteristics [8]. There is currently no TKR specific mortality prediction model.

A well performing robust model that predicts mortality could be used to aid in decision making for TKR as well as targeting interventions for high risk patients. As such the hypothesis of this study is that 90-day all-cause mortality is predictable using routinely collected data. This will be assessed by developing and externally validating a model using area under receiver operator curve.

## Materials and methods

This retrospective cohort study used observational healthcare databases from the UK (The Health Improvement Network (THIN) [3]) and US (Optum). Detailed information on these databases is available in Table 1. All databases used in this paper were mapped into the Observational Medical Outcomes Partnership Common Data Model (OMOP-CDM) [11]. The OMOP-CDM was developed for researchers to transform diverse datasets into a consistent structure and vocabulary. This means studies using these databases are more replicable increasing the clinical relevance of evidence.

**Table 1:** Database Information

Database	Database acronym	Country	Data type	Time period
Optum© De-Identified Clinformatics^®^ Data Mart Database	ClinFormatics	US	Claims	2000–2018
IQVIA Medical Research Data ([IMRD], incorporating data from The Health Improvement Network [THIN]	THIN	UK	General practice	2003–2018

Each site obtained institutional review board approval for the study or used de-identified data and therefore the study was determined not to be human subjects research. Informed consent was not necessary at any site.

### Cohorts

#### Development target population cohort

The target population for model development and validation contained patients with knee osteoarthritis undergoing TKR. The first recorded TKR procedure identified was considered the event of interest with the date of surgery as index date. Inclusion criteria required patients to have at least 1 year of continuous pre-index date recorded observation time. Individuals below the age of 40, those with prior evidence of knee arthroplasty, knee fracture, knee surgery (except diagnostic procedures), rheumatoid arthritis, inflammatory arthropathies, or septic arthritis at any time before the index date were excluded. This is because these patients likely have a cause other than osteoarthritis for their surgery. Patients with spine, hip, or foot pathology observed in the 365 days before index date were also excluded.

The target cohort for TKR is available at: TKR: http://atlas-demo.ohdsi.org/#/cohortdefinition/1776551.

#### Outcome cohorts

Mortality was defined as all-cause mortality based on records of date of death. This is well captured in THIN and in Optum until 2013, when a change in reporting means that the capture after this time is specific but less sensitive. Available at: http://atlas-demo.ohdsi.org/#/cohortdefinition/1776555.

Patients were considered at risk for mortality from the day after surgery up until day 90.

### Candidate predictors

89,031 candidate predictors were derived from the observational healthcare data that existed on or prior to the target index date (TKR surgery date). These variables were demographics, binary indicators of medical events (e.g. GP visit, disease diagnosis, medication prescription) and counts of record types. The demographics were gender, 5 year age groups (40–45, 45–50,…,95+) and month of the target index date. Binary indicator variables for medical events were created based on the presence or absence of each concept for a patient corresponding to the OMOP-CDM clinical domains of conditions, drugs, procedures or measurements. For conditions binary predictors were created using the 30 days and 365 days prior to index date. For example, there exists one covariate for each of ‘Diabetes mellitus’, ‘Hypertensive disorder’, and ‘Hypercholesterolemia’ (and similarly for other diseases that appear in the patient records), based on the occurrence of a diagnosis code for each condition in the 365 days or 30 days preceding the index date. Drug covariates were constructed similarly, but used time windows of 30, 365, 1095 days and all time prior to target index date. Covariates representing counts of how many visits (e.g. primary care visit) a patient had in the 365 days and 1095 days prior to the target index date were also created. The following existing risk scores (CHADS2, CHA2DS2VASc (both stroke risk models), Diabetes Complications severity index, Charlson Comorbidity Index) using all data prior to index were also calculated and used as candidate predictors.

### Methodology for model development and validation

The study was initially conducted using the THIN and OPTUM datasets. Models predicting the 90-day mortality in the TKR target population were developed in both databases. The interoperability of the OMOP-CDM was utilised to externally validate in the non-development database.

Model development followed the framework for the creation and validation of patient-level prediction (PLP) models presented in Reps et al. [13], a person ‘train-test split’ method was used to perform internal validation. In each development cohort, the random split sample (`training sample’) containing 75% of patients was used to develop the prediction models and the remaining 25% of patients (`test sample’) was used to validate the risk scores. The models were trained using least absolute shrinkage and selection operator (LASSO) regularised logistic regression, using a threefold cross validation technique in the training sample to learn the optimal regularisation hyper-parameter through an adaptive search [16]. LASSO regularization [17] helps to limit overfitting in model development. This process works by assigning a “penalty” to the inclusion of a variable, this variable must then contribute more to the performance than the penalisation. If this condition is not met then the coefficient of the covariate becomes 0, which eliminates the covariate from the model, thus automating feature selection.

Performance of the model was assessed in terms of discrimination and calibration. Discrimination assesses how well the model can distinguish which patients experience the outcome and calibration assesses whether the predicted risks are in alignment with the observed risks. Discrimination was measured using the Area Under Receiver Operator Characteristic Curve (AUROC). An AUROC of greater than 0.70 is considered to be a reasonable candidate for external validation. The model calibration was assessed by plotting the predicted and observed risks across deciles of predicted risk. Calibration assessment is then performed visually rather than using a statistic or numeric value as this provides an impression of the direction and scale of miscalibration [7]. Summary statistics were reported from the test samples.

External validation [14] was performed by applying the final prediction models in the dataset not used for development. The external validation was analysed in the same way as internally.

### Model parsimonisation

When using a data-driven approach to model development, generally the final models contain a large number of covariates. The full model assesses what is in principle the best possible performing model. However, the large number of covariates can create a barrier to implementation and understanding.

Models were therefore created that could be candidates for the clinical implementation by performing further analyses to reduce the number of features in the final model (improving parsimony). This analysis investigated what the performance loss is when using fewer covariates.

The approach involved analysing the covariates selected by the final model and then using clinical expertise to attempt to combine multiple of these covariates that correspond to a similar illness, into a single covariate. Often, LASSO logistic regression models include multiple covariates which are clinically related, for example a model might select the same condition occurrence but in different time periods predating the index date (e.g. ‘diabetes − 30 days to 0 days prior to index’ and ‘diabetes 365 days to 0 days prior to index’). These could be simplified to an aggregate covariate of “History of Diabetes”, rather than multiple covariates specifying the specific time frame of the occurrence.

The procedures for developing both the full and parsimonious models will be identical except for the covariates. Definitions of the aggregated covariates are available in Appendix 2.

All statistical analysis was performed using R (version 3.5.1) and the Patient-Level Prediction. This study was conducted and reported according to the Transparent Reporting of a multivariate prediction model for Individual Prediction or Diagnosis (TRIPOD) guidelines [10]. All the analysis code used for the development of the models is available on github at https://github.com/OHDSI/StudyProtocolSandbox/tree/master/mortalityValidation as well as the developed mortality models themselves for external validation at: https://github.com/ohdsi-studies/TkrPredictSimple.

## Results

The target population included 40,950 (THIN) and 152,665 (Optum) patients. 90-day mortality occurred in 0.20% (THIN)–0.23% (Optum) of patients (Table 2).

**Table 2:** Performance and population sizes for the mortality models

Dataset	Target population	90-Day mortality	
		Size	AUROC
OPTUM	152,665	353 (0.23%)	0.78
THIN	40,950	81 (0.20%)	0.68

The 90-day mortality model trained using OPTUM obtained internal AUROC above 0.7 (Table 2). The external validation of the 90-day mortality models developed on OPTUM and THIN ranged between 0.68 and 0.86 and are presented in Table 2. Details of the distribution of key covariates can be found in Appendix 1.

The OPTUM 90-day mortality model performed better than the THIN 90-day mortality model both internally and across the external validation (Table 2). The OPTUM 90-day mortality model achieved a slightly increased performance (AUROC 0.69) in the THIN dataset compared to the internal validation of the THIN developed model (AUROC 0.68). For the 90-day mortality OPTUM model, 102 of 89,031 candidate variables were selected into the final model. The full model is available in Appendix 3.

The models and performance on the test and external validation sets are available to explore interactively at http://data.ohdsi.org/TKROutcomesExplorer/.

The prevalence of a selection of covariates included in the 90-day mortality model developed using OPTUM, when assessed in multiple databases can be found in Appendix 1.

This analysis shows that the covariate prevalence varies between the different databases, suggesting the databases have different underlying characteristics. As the models maintain performance despite these differences, it suggests that the model is robust to variability in the distribution of the covariates.

The 90-day Optum mortality was then parsimonised. The creation of these aggregate covariates and their definitions are available in Appendix 2. This model is detailed in Table 3.

**Table 3:** Parsimonious model with covariates and coefficients for predicting 90-day mortality following TKR

Covariate	Value
Intercept	− 6.64376
*Age group*	
40–44	− 4.40718
45–49	− 5.72523
50–54	− 0.61149
55–59	− 0.25853
60–64	− 0.21392
65–69	− 0.01862
70–74 (reference)	0
75–79	0.60808
80–84	1.08846
85–89	1.88595
90–94	− 1.42352
*Gender*	
Male	0.36173
Female (reference)	0
*History of*	
Cancer (excl non-melanoma skin cancer)	− 0.21177
COPD	0.44467
Gout	0.45821
Heart failure or atrial fibrillation	1.25532
Hypertension	− 0.12567
Kidney disease	0.5571
OA	− 0.4513
T2DM	0.27827
Opioid use	− 0.35781
Psycholeptics use	0.17227

**Fig. 1:**
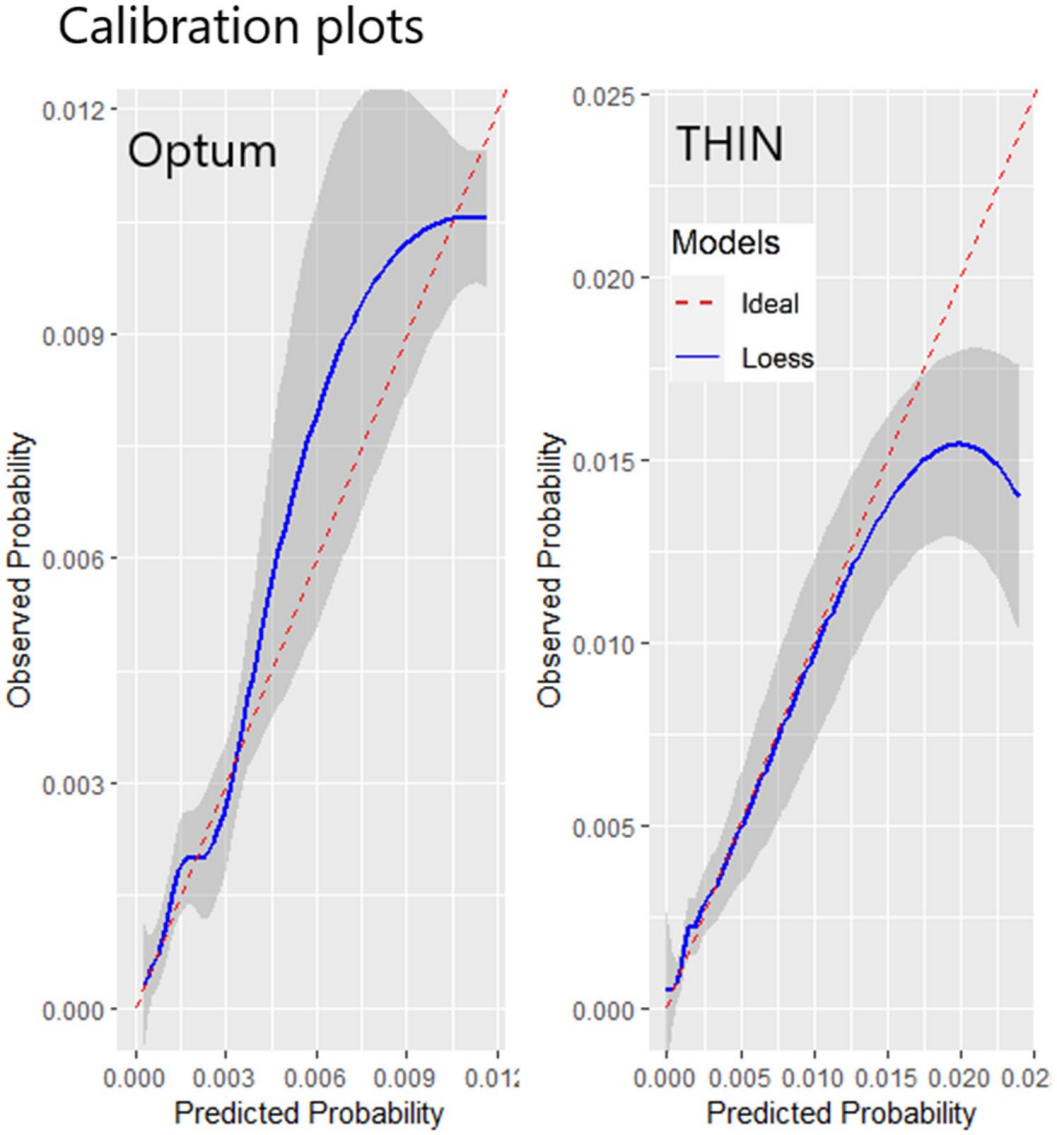
Calibration plot showing the calibration of the parsimonious model internally (Optum) and externally (THIN). The plot shows the agreement between the observed and predicted risk for patients. This is calculated by fitting loess regression

When the analysis was performed with these covariates, the AUROC was 0.77 internally and 0.71 in THIN. The results are available in Table 4. The calibration plot for the internal validation and the THIN validation are presented in Fig. 1. Figure 1 shows that, for the majority of patients, the model is well calibrated internally with the ideal line always appearing within the confidence interval. For the external validation in THIN, the model is well calibrated however for patients at higher risk there is some overestimation of risk in the highest risk groups. For example, a predicted risk of 0.02 corresponds to an observed risk of 0.015. The model could potentially benefit from recalibration in this setting.

## Discussion

The main finding of this study is the predictability of post-operative 90-day mortality following TKR. The AUROC of LASSO logistic regression model was found to be 0.78 in the OPTUM database. Validating this model against the other databases resulted in AUROC values of 0.68 (THIN) indicating that the model is fairly robust. The high number of features (102) in this model presents a barrier to implementation in clinics. A parsimonious model was therefore created, containing 12 variables. This model achieved AUROC of 0.77 in the training data and 0.71 in the external validation in the THIN database. The calibration was adequate although there appeared to be an overestimation of risk for patients at higher risk when assessed in THIN. As the parsimonious model achieved similar or better performance and is more implementable, it is preferred.

The desired operating characteristics when applying the parsimonious OPTUM 90-day mortality model to classify patients into those who will die and those who will not within 90 days of the surgery can be picked based on the prediction threshold, see Table 3. As an example, if a female patients aged 75 presented to a clinician whilst she had COPD and T2DM, then her raw score would be

$$-6.64376 \left(\mathrm{intercept}\right)+ 0.60808 \left(\mathrm{age}=75\right)+ 0.44467 \left(\mathrm{COPD}\right)+ 0.27827 \left(\mathrm{T}2\mathrm{DM}\right)= -5.31274.$$

Which maps to a predicted risk of 0.5%. When compared to the outcome prevalence of 0.2% this shows the patient is twice as likely as average to die following this surgery.

**Table 4:** Internal and external validations of the full and parsimonious (reduced) feature models

Development database	Validation database	Model type	AUROC	Test population	Outcome count in test population (incidence in cases per 100 patients)
OPTUM	OPTUM	Full	0.78	38,166	88 (0.23)
OPTUM	THIN	Full	0.70	57,897	121 (0.30)
THIN	THIN	Full	0.68	10,237	20 (0.20)
OPTUM	OPTUM	Reduced	0.77	38,157	88 (0.23)
OPTUM	THIN	Reduced	0.71	57,897	121 (0.30)
THIN	OPTUM	Full	0.68	152,665	353 (0.23)

In contrast to previous studies, the focus of this research was to develop the best performing predictive model on basis of all clinical and demographic data recorded in the observational databases and to then assess how close to this performance a reduced feature set model could come. The predictors included in the final model were mostly already known to be related to the outcome, what this study adds is to provide a quantitative relationship between the combination of these and the probability of the outcome. This was done by performing a regression analysis using these covariates. The selection of these predictors speaks to the robustness of the methods. Previous prediction models in the context of knee replacement have focussed on patient-reported outcomes or revision surgery/implant survivorship, with little focus on complications or post-operative mortality, meaning comparison to these is difficult [1]. When considering common mortality predictors such as the American College of Surgeons National Surgical Quality Improvement Programme comparisons are difficult using observational data as “Functional status” is not well captured in observational studies. Further, the Revised Cardiac Risk Index generally performs with a median AUROC of 0.62 showing lower performance than the model developed in this study [4].

Hunt et al. report an incidence of mortality (0.37%) in their study on 45-day mortality following knee replacement surgery [5]. This is high compared with our reported incidence of mortality, which could be due to the limitation of the mortality capture in the databases studied. The low incidence of death (around 0.2%) following TKR necessitates large datasets with accurate recording of mortality. The reported 90-day mortality predictive model may be used as a complementary element for screening of high-risk patients and better preparation before surgery. It could also allow the patient and clinician to be better informed about the potential benefit-risk of elective TKR. Given that all-cause mortality was considered, the mortality is not necessarily caused by the TKR, however if the patient is deemed to be at a high risk of mortality in the 90-day post-operative period then the surgery is still likely inadvisable due to the costs to both the patient and the healthcare system without providing benefit.

Limitations of this study include the low number of outcomes in some of the analyses meaning that estimates are potentially unreliable, as well as potential misclassification of covariates in the data. The recording of death in the THIN database is very reliable but in Optum is known to be specific but lacking some sensitivity because in 2013 reporting of death stopped being mandatory. This could lead to an underestimation of the number of deaths following a TKR in this study. Further limitations are that although large numbers of covariates are included in the analysis, some covariates are poorly captured in the data used. Known predictors such as surgeon skill and volume are not available in routinely collected healthcare data and as such have not been included. As with all observational studies, the models can only be assessed on the predictors available and as such any predictors which are not in the source data, will be missed by the models.

Limitations of the phenotypes include: (1) there is a potential contamination issue in the TKR cohort as prior to ICD-10 coding, TKR cohorts will have UKR cases as the same ICD procedure code was valid for both (2) if a patient were to have bilateral TKR only the first surgery would be included in our target cohort and the second would be excluded.

A major strength of this study is that the model is already externally validated, demonstrating its robustness and transportability, a process typically taking 3-years (12). The low number of features of this model is a significant advantage to implementation.

## Conclusion

In conclusion, a model was developed and externally validated for 90-day mortality after a TKR. This prediction model has both good discrimination performance and calibration which was maintained across the external validation. Thus, this model is a strong candidate for impacting clinical decision making.

## Supplementary Information

Below is the link to the electronic supplementary material.

Supplementary file1 (XLSX 19 kb)

Supplementary file2 (DOCX 38 kb)

Supplementary file3 (DOCX 26 kb)

## Abbreviations

TKR: Total knee replacement
OA: Osteoarthritis
OMOP-CDM: Observational Medical Outcomes Partnership Common Data Model
EHR/EMR: Electronic health/medical records
GP: General practitioner
AUROC: Area under receiver operator characteristic curve
TRIPOD: Transparent reporting of a multivariate prediction model for individual prediction or diagnosis
EHDEN: European Health Data and Evidence Network

## References

[1] Arden N, Altman D, Beard D, Carr A, Clarke N, Collins G et al (2017) Lower limb arthroplasty: Can we produce a tool to predict outcome and failure, and is it cost-effective? An epidemiological study. Programme Grants Appl Res 5(12) 28678462

[2] Berstock JR, Beswick AD, Lopez-Lopez JA, Whitehouse MR, Blom AW (2018). Mortality after total knee arthroplasty: a systematic review of incidence, temporal trends, and risk factors. J Bone Jt Surg Am.

[3] Blak BT, Thompson M, Dattani H, Bourke A (2011). Generalisability of The Health Improvement Network (THIN) database: demographics, chronic disease prevalence and mortality rates. Inform Prim Care.

[4] Ford MK, Beattie WS, Wijeysundera DN (2010). Systematic review: prediction of perioperative cardiac complications and mortality by the revised cardiac risk index. Ann Intern Med.

[5] Hunt LP, Ben-Shlomo Y, Clark EM, Dieppe P, Judge A, MacGregor AJ (2014). 45-day mortality after 467,779 knee replacements for osteoarthritis from the National Joint Registry for England and Wales: an observational study. Lancet.

[6] Inacio MCS, Pratt NL, Roughead EE, Graves SE (2016). Evaluation of three co-morbidity measures to predict mortality in patients undergoing total joint arthroplasty. Osteoarthr Cartil.

[7] Iqbal J, Vergouwe Y, Bourantas CV, van Klaveren D, Zhang YJ, Campos CM (2014). Predicting 3-year mortality after percutaneous coronary intervention: updated logistic clinical SYNTAX score based on patient-level data from 7 contemporary stent trials. JACC Cardiovasc Interv.

[8] Konopka JF, Hansen VJ, Rubash HE, Freiberg AA (2015). Risk assessment tools used to predict outcomes of total hip and total knee arthroplasty. Orthop Clin N Am.

[9] Kunutsor SK, Whitehouse MR, Blom AW, Beswick AD (2017). Systematic review of risk prediction scores for surgical site infection or periprosthetic joint infection following joint arthroplasty. Epidemiol Infect.

[10] Moons KG, Altman DG, Reitsma JB, Ioannidis JP, Macaskill P, Steyerberg EW (2015). Transparent Reporting of a multivariable prediction model for Individual Prognosis or Diagnosis (TRIPOD): explanation and elaboration. Ann Intern Med.

[11] Overhage JM, Ryan PB, Reich CG, Hartzema AG, Stang PE (2012). Validation of a common data model for active safety surveillance research. J Am Med Inform Assoc.

[12] Pearse RM, Moreno RP, Bauer P, Pelosi P, Metnitz P, Spies C (2012). Mortality after surgery in Europe: a 7 day cohort study. Lancet.

[13] Reps JM, Schuemie MJ, Suchard MA, Ryan PB, Rijnbeek PR (2018). Design and implementation of a standardized framework to generate and evaluate patient-level prediction models using observational healthcare data. J Am Med Inform Assoc.

[14] Reps JM, Williams RD, You SC, Falconer T, Minty E, Callahan A (2020). Feasibility and evaluation of a large-scale external validation approach for patient-level prediction in an international data network: validation of models predicting stroke in female patients newly diagnosed with atrial fibrillation. BMC Med Res Methodol.

[15] Springer BD, Cahue S, Etkin CD, Lewallen DG, McGrory BJ (2017). Infection burden in total hip and knee arthroplasties: an international registry-based perspective. Arthroplasty Today.

[16] Suchard MA, Simpson SE, Zorych I, Ryan P, Madigan D (2013). Massive parallelization of serial inference algorithms for a complex generalized linear model. ACM Trans Model Comput Simul.

[17] Tibshirani R (1996). Regression shrinkage and selection via the Lasso. J R Stat Soc Series B Stat Methodol.

