# Supplementary material for: 90-Day all-cause mortality can be predicted following a total knee replacement: an international, network study to develop and validate a prediction model
Source: Knee Surg Sports Traumatol Arthrosc. 2021 Dec 6;30(9):3068–75. doi: 10.1007/s00167-021-06799-y (PMC9418076; doi:10.1007/s00167-021-06799-y)
Supplement: Supplementary file 2 — Supplementary file2 (DOCX 38 kb) [file 167_2021_6799_MOESM2_ESM.docx]

# Appendix 2: Parsimonious model derivation

To go from the data-driven model to the model with reduced variables we implemented this process for each of the scores.

1. A clinician inspected the data-driven model to identify variables that had a high standardized mean difference between those patients with and without the outcome. There are often multiple predictors which are related and correlated selected by the model, for example a model might select as associated a condition occurrence in different time periods predating the index date. This could be simplified to a predictor saying only “Patient had condition X in history”, rather than multiple predictors specifying within which time period the condition occurred, or multiple codes that are probably related to a specific condition. We identified general categories from these such as ‘heart disease’ and ‘diabetes’.
2. Phenotype definitions for each category were created.
3. We trained a LASSO logistic regression model on the original data using age groups, sex and the newly created predictors indicating whether the patient had any of the category predictors.
4. This gave us the parsimonious model.

Covariate Descriptions

In the Observational Medical Outcomes Partnership Common Data Model (OMOP-CDM) diagnoses are classified in hierarchies. This means that a certain diagnosis, or condition occurrence in the context of this document, has a number of descendants. Diabetes mellitus, for example, is a descendant of diabetes and has numerous, more detailed descendants such as type 1 diabetes mellitus, which can be illustrated like this:


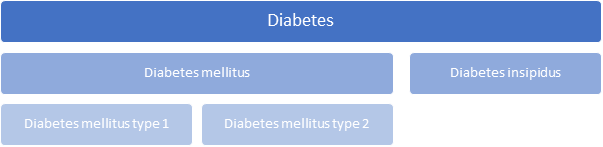


The actual hierarchical structure of SNOMED-CT, one of the classifications on which the OMOP-CDM rests, is more complex but the notion remains.

For most diseases, we cannot know the exact time of onset, and must use the point in time on which the condition was recorded (condition occurrence) as the proxy. Thus, within each of the following groups, a history of the condition in question is operationalised as at least one occurrence of the listed conditions at any time prior to index. The concept id's uniquely identify conditions and are made as hyperlinks taking the reader to the ATHENA interface (https://athena.ohdsi.org/) facilitating perusal of their hierarchical positions.

| **Condition occurrences** | **Examples** | **Concept id’s** |
| --- | --- | --- |
| \| History of cancer \| \| --- \| \| Any malignant neoplastic disease or descendants \| \| Except squamous cell carcinoma \| \| Except basal cell carcinoma \| | \| Leukaemia  Large-cell carcinoma in lung \| \| --- \| \|  \| | \| [443392](https://athena.ohdsi.org/search-terms/terms/443392/graph?levels=4&standardsOnly=true&zoomLevel=4) \| \| --- \| \| [4111921](https://athena.ohdsi.org/search-terms/terms/4111921/graph?levels=4&standardsOnly=true&zoomLevel=4) \| \| [4112752](https://athena.ohdsi.org/search-terms/terms/4112752/graph?levels=4&standardsOnly=true&zoomLevel=4) \| |
| History of COPD |  |  |
| Chronic obstructive pulmonary disease or descendants | Pulmonary emphysema  Acute exacerbation of COPD | [255573](https://athena.ohdsi.org/search-terms/terms/255573/graph?levels=4&standardsOnly=true&zoomLevel=4) |
| History of Gout | Gout  Anti Gout treatment | 440674  21604128 |
| History of Heart Failure or Atrial Fibrillation | Congestive Heart Failure  Chronic Atrial Fibrillation | 319835  4141360 |
| History of hypertension  Hypertension or any descendants | Essential hypertension  Benign hypertension | [316866](https://athena.ohdsi.org/search-terms/terms/316866/graph?levels=4&standardsOnly=true&zoomLevel=4) |
|  |  |  |
| History of kidney disease  Kidney disease or descendants. We consider  both chronic and acute kidney  disease together for a broader cohort  definition. | Acute nephropathy  Hypertensive renal disease | [198124](https://athena.ohdsi.org/search-terms/terms/198124/graph?levels=4&standardsOnly=true&zoomLevel=4) |
| History of Osteo-arthritis | Spondylosis | 4227449 |
| History of Opioid use | Prescription for Hydrocodone | 1174888 |
| History of Psycholeptics use | Prescription for Perphenazine | 733008 |
| \| History of Type 2 diabetes mellitus \| \| --- \| \| Diabetes mellitus (Type 2) or descendants \| \| Documented poor glycaemic control with/without complications \| \| Complication due to diabetes mellitus \| | \|  \| \| --- \| \| Type 2 diabetes mellitus \| \|  \| | [443238](https://athena.ohdsi.org/search-terms/terms/443238/graph?levels=4&standardsOnly=true&zoomLevel=4) |

The phenotypes for each Parsimonious predictor are available in in the Github package.
