# Supplementary material for: 90-Day all-cause mortality can be predicted following a total knee replacement: an international, network study to develop and validate a prediction model
Source: Knee Surg Sports Traumatol Arthrosc. 2021 Dec 6;30(9):3068–75. doi: 10.1007/s00167-021-06799-y (PMC9418076; doi:10.1007/s00167-021-06799-y)
Supplement: Supplementary file 3 — Supplementary file3 (DOCX 26 kb) [file 167_2021_6799_MOESM3_ESM.docx]

**3. Optum trained 90-day Mortality model**

| Covariate | Coefficient |
| --- | --- |
| Female (reference) | 0 |
| Male | 0.032433 |
| age group: 75-79 | 0.056367 |
| age group: 80-84 | 0.764245 |
| age group: 85-89 | 1.21805 |
| History of Acute infectious disease | -0.02355 |
| History of Arthralgia of the pelvic region and thigh | 0.023708 |
| History of Chest injury | 0.032244 |
| History of Chronic obstructive lung disease | 0.054521 |
| History of Conduction disorder of the heart | 0.019192 |
| History of Contusion | 0.067503 |
| History of Degenerative disorder of musculoskeletal system | -0.05592 |
| History of Diabetes mellitus | 0.193733 |
| History of Dizziness | -0.20057 |
| History of Edema | 0.082884 |
| History of Inflammation of specific body systems | -0.00752 |
| History of Mass of lower limb | -0.12112 |
| History of Mass of soft tissue | -0.03481 |
| History of Mitral valve disorder | 0.096638 |
| History of Pain in upper limb | -0.1443 |
| History of Pain of cardiovascular structure | -0.10054 |
| History of Respiratory insufficiency | 0.432687 |
| History of Retinal disorder | 0.062518 |
| History of Senile hyperkeratosis | -0.01732 |
| History of Tricuspid valve disorder, non-rheumatic | 0.080205 |
| History of Vascular disorder | 0.055287 |
| History of Vitamin disease | -0.06581 |
| Diagnosis in last 3 years of Atrial arrhythmia | 0.003079 |
| Diagnosis in last 3 years of Atrial fibrillation | 0.022018 |
| Diagnosis in last 3 years of Benign essential hypertension | 0.353412 |
| Diagnosis in last 3 years of Chronic disease of respiratory system | 0.007 |
| Diagnosis in last 3 years of Chronic obstructive lung disease | 0.018677 |
| Diagnosis in last 3 years of Congestive heart failure | 0.095554 |
| Diagnosis in last 3 years of Deficiency of micronutrients | -0.00465 |
| Diagnosis in last 3 years of Degenerative disorder of musculoskeletal system | -0.02798 |
| Diagnosis in last 3 years of Fibrillation | 0.078604 |
| Diagnosis in last 3 years of Gout | 0.020712 |
| Diagnosis in last 3 years of Heart failure | 0.512081 |
| Diagnosis in last 3 years of Inflammatory disorder of head | -0.07688 |
| Diagnosis in last 3 years of Interstitial lung disease | 0.036129 |
| Diagnosis in last 3 years of Lesion of eye structure | -0.1393 |
| Diagnosis in last 3 years of Mass of trunk | -0.15194 |
| Diagnosis in last 3 years of Neuropathy | -0.0192 |
| Diagnosis in last 3 years of Respiratory obstruction | -0.00568 |
| Diagnosis in last 3 years of Soft tissue lesion | -0.00908 |
| Diagnosis in last 3 years of Vitamin disease | -0.10386 |
| Diagnosis in last 30 days of Chronic disease of respiratory system | 0.158373 |
| Diagnosis in last 30 days of Degenerative disorder of musculoskeletal system | -0.01892 |
| Diagnosis in last 30 days of Heart failure | 0.020969 |
| Diagnosis in last 30 days of Pain finding at anatomical site | -0.0234 |
| Diagnosis in last 365 days of Chronic obstructive lung disease | 0.183538 |
| Diagnosis in last 365 days of Degenerative disorder of musculoskeletal system | -0.29977 |
| Diagnosis in last 365 days of Heart disease | 0.181488 |
| Diagnosis in last 365 days of Heart failure | 0.212408 |
| Diagnosis in last 365 days of Kidney disease | 0.155339 |
| Diagnosis in last 365 days of Malaise | -0.06607 |
| Diagnosis in last 365 days of Measurement finding below reference range | 0.056003 |
| Diagnosis in last 365 days of Peripheral vascular disease | 0.071121 |
| Diagnosis in last 365 days of Soft tissue lesion | -0.01555 |
| Diagnosis in last 365 days of Vascular disorder | 0.102196 |
| Prescription in all time prior for ANTIDIARRHEALS, INTESTINAL ANTIINFLAMMATORY/ANTIINFECTIVE AGENTS | 0.06478 |
| Prescription in all time prior for ANTIGOUT PREPARATIONS | 0.009398 |
| Prescription in all time prior for ANTIGOUT PREPARATIONS | 0.011559 |
| Prescription in all time prior for ANTIINFLAMMATORY AND ANTIRHEUMATIC PRODUCTS | -0.10029 |
| Prescription in all time prior for BETA-LACTAM ANTIBACTERIALS, PENICILLINS | -0.06451 |
| Prescription in all time prior for Diltiazem | 0.100664 |
| Prescription in all time prior for Furosemide | 0.058481 |
| Prescription in all time prior for MACROLIDES, LINCOSAMIDES AND STREPTOGRAMINS | -0.04145 |
| Prescription in all time prior for Natural opium alkaloids | -0.07518 |
| Prescription in all time prior for PSYCHOLEPTICS | 0.113607 |
| Prescription in all time prior for Thyroid hormones | -2.57E-06 |
| Prescription in all time prior for THYROID PREPARATIONS | -0.00453 |
| Prescription in last 3 years for Benzothiazepine derivatives | 0.005689 |
| Prescription in last 3 years for DERMATOLOGICALS | -0.00012 |
| Prescription in last 3 years for Diltiazem | 0.060977 |
| Prescription in last 3 years for Hyaluronan | -0.1619 |
| Prescription in last 30 days for ALIMENTARY TRACT AND METABOLISM | 0.027216 |
| Prescription in last 30 days for Furosemide | 0.093061 |
| Prescription in last 365 days for CALCIUM CHANNEL BLOCKERS | 0.053113 |
| Prescription in last 365 days for CARDIAC THERAPY | 0.13619 |
| Prescription in last 365 days for Furosemide | 0.106493 |
| Prescription in last 365 days for Potassium Chloride | -0.04889 |
| Prescription in last 365 days for RESPIRATORY SYSTEM | -0.19104 |
| Prescription in last 365 days for SENSORY ORGANS | 0.042467 |
| Measurement in previous 365 days of Erythrocyte mean corpuscular hemoglobin concentration [Mass/volume] by Automated count | -0.0811 |
| Measurement in previous 365 days of Urinalysis, by dip stick or tablet reagent for bilirubin, glucose, hemoglobin, ketones, leukocytes, nitrite, pH, protein, specific gravity, urobilinogen, any number of these constituents; automated, with microscopy | -0.13782 |
| Adult health examination in previous 365 days | -0.01514 |
| Procedure performed in all time prior of Arthrocentesis, aspiration and/or injection, major joint or bursa (eg, shoulder, hip, knee, subacromial bursa); without ultrasound guidance | 0.01068 |
| Procedure performed in all time prior of Collection of venous blood by venipuncture | -0.02251 |
| Procedure performed in all time prior of Extracapsular cataract removal with insertion of intraocular lens prosthesis (1 stage procedure), manual or mechanical technique (eg, irrigation and aspiration or phacoemulsification) | 0.19466 |
| Procedure performed in all time prior of Gynecologic examination | -0.09535 |
| Procedure performed in all time prior of Hospital discharge day management; more than 30 minutes | 0.116508 |
| Procedure performed in all time prior of Office or other outpatient visit for the evaluation and management of a new patient, which requires these 3 key components: A detailed history; A detailed examination; Medical decision making of low complexity. Counseling and/or coordination of care with | -0.05792 |
| Procedure performed in all time prior of Periodic comprehensive preventive medicine reevaluation and management of an individual including an age and gender appropriate history, examination, counseling/anticipatory guidance/risk factor reduction interventions, and the ordering of laboratory/diag | -0.22667 |
| Procedure performed in all time prior of Pre-surgery evaluation | 0.027928 |
| Procedure performed in all time prior of Procedure with a procedure focus | -0.19325 |
| Procedure performed in all time prior of Screening mammography | -0.09322 |
| Procedure performed in all time prior of Subsequent hospital care, per day, for the evaluation and management of a patient, which requires at least 2 of these 3 key components: A detailed interval history; A detailed examination; Medical decision making of high complexity. Counseling and/or coor | 0.034488 |
| Procedure performed in all time prior of Subsequent hospital care, per day, for the evaluation and management of a patient, which requires at least 2 of these 3 key components: An expanded problem focused interval history; An expanded problem focused examination; Medical decision making of moder | 0.004933 |
| Procedure performed in all time prior of Unlisted diagnostic radiographic procedure | -0.15888 |
| Procedure performed in previous 365 days of Office or other outpatient visit for the evaluation and management of a new patient, which requires these 3 key components: A detailed history; A detailed examination; Medical decision making of low complexity. Counseling and/or coordination of care with | -0.18707 |
| Procedure performed in previous 365 days of Radiologic examination, chest, 2 views, frontal and lateral | 0.016066 |
| Procedure performed in previous 365 days of Radiologic examination, knee; complete, 4 or more views | -0.02786 |
